# An innovative method to determine the stress-dependency of Poisson’s ratio of granitic rocks

**DOI:** 10.1038/s41598-024-75892-2

**Published:** 2025-05-08

**Authors:** Samad Narimani, László Kovács, Balázs Vásárhelyi

**Affiliations:** 1 Department of Engineering Geology and Geotechnics, Budapest University of Technology and Economics, Budapest, Hungary; 2 RockStudy Ltd. (Komero Kft.), U7633 Pecs, Hungary

**Keywords:** UCS, Poisson’s ratio, Granitic rock, Crack initiation stress, Crack damage stress, Civil engineering, Mathematics and computing

## Abstract

Uniaxial Compressive test (UCS) results are essential in evaluation the values of Poisson’s ratio. However, according to the suggestion of the International Society for Rock Mechanics, Poisson’s ratio can be determined using three alternative methods: the secant, average and tangent. Applying these methods causes discrepancies in the results; according to our experiences, the differences can be threefold or more. This paper aims to study the process of changes of Poisson’s ratio for intact rock during loading from micro-crack initiation to failure stage. The objective of this theoretical investigation is to establish a straightforward mathematical formulation between σ/σ_c_ and intact rock’s Poisson’s ratio value. To outline these changes forty two granite rocks were investigated from Bátaapáti radioactive waste repository (Hungary) and the calculation was performed by using the new formula from the beginning of loading till failure stage at UCS test. In the laboratory test program, Poisson’s ratio derived from standard tests varies with momentary stress; it steadily increases as stress rises until reaching the stress level causing unstable crack propagation. Additionally, the Poisson’s rate follows a linear increase with stress, up to the point of unstable crack propagation stress. The research demonstrated that the proposed equations provide competent values for the root mean squared error value (ranging from 0 to 0.04), the mean absolute percentage error (ranging from 0.6% to 18%) and the mean absolute error (ranging from 0 to 0.04). Contrary to previous ideas, our results suggest that the Poisson’s ratio is not a constant for rigid rocks.

## Introduction

In the elastic deformation of rocks and rock masses subjected to static or dynamic loads, Poisson’s ratio, which describes the relationship between the orthogonal deformations, plays an unquestionably significant role. Additionally, its impacts can be seen in a wide range of rock engineering applications, from straightforward laboratory testing on intact specimens to on-site measurements of in situ stresses or the deformability of rock masses. Rock engineering can therefore benefit from knowledge of various Poisson’s ratio features. Researchers will be able to conduct more accurate laboratory and in-situ studies and gain a deeper understanding of the mechanisms underlying rock (mass) deformation if they have a thorough understanding of Poisson’s ratio^1–3^.

Due to the more complex behavior of both intact rocks and real rock masses the traditional approaches of technical mechanics often fail. Throughout the compaction stage, the Poisson’s ratio can be distorted by the various flaws, internal fractures, and pores found in rocks due to their different mineral composition^1,4,5^. In spite of the fact that Poisson’s ratio is an essential variable in theoretical investigations and numerical simulations, there are little information on its evaluation, testing, and application range. Without fully understanding the significance of determining Poisson’s ratio of rocks scientifically, engineers and researchers still frequently consider it as a material constant in everyday static design tasks, both in analytical and numerical methods, although its stress-dependent character has been proven in innumerable experiments^1,5^. At the failure stage of rock cracking, however, Poisson’s ratio shows a considerable reliance on stress because of the nonlinear growth of plastic deformation^6,7^. According to Fan et al.^8^, there is a faster nonlinear expansion of the lateral strain than the axial strain with rising axial stress. Additionally, the tangent Poisson’s ratio grows with stress levels, finally surpassing 0.5.

In accordance with Bieniawski^9^, the Poisson’s ratio of rocks remains constant during linear elastic deformation but starts to rise as a result of the emergence of new microcracks or the growth of preexisting ones^9^. According to Gercek’s research^2^ and the advice of the American Association of State Highway and Transportation Office^10^, typical ranges of values for the Poisson’s ratio of a few rock types have been compiled. Due to Gercek^2^, the Poisson’s ratio is an elastic constant whose relevance is typically underestimated when compared to other fundamental mechanical characteristics of rocks. There are many different aspects of rock mechanics that call for prior understanding or an estimate of the Poisson’s ratio’s value. His study focused on the rock mechanics Poisson’s ratio values and applications.

**Fig. 1:**
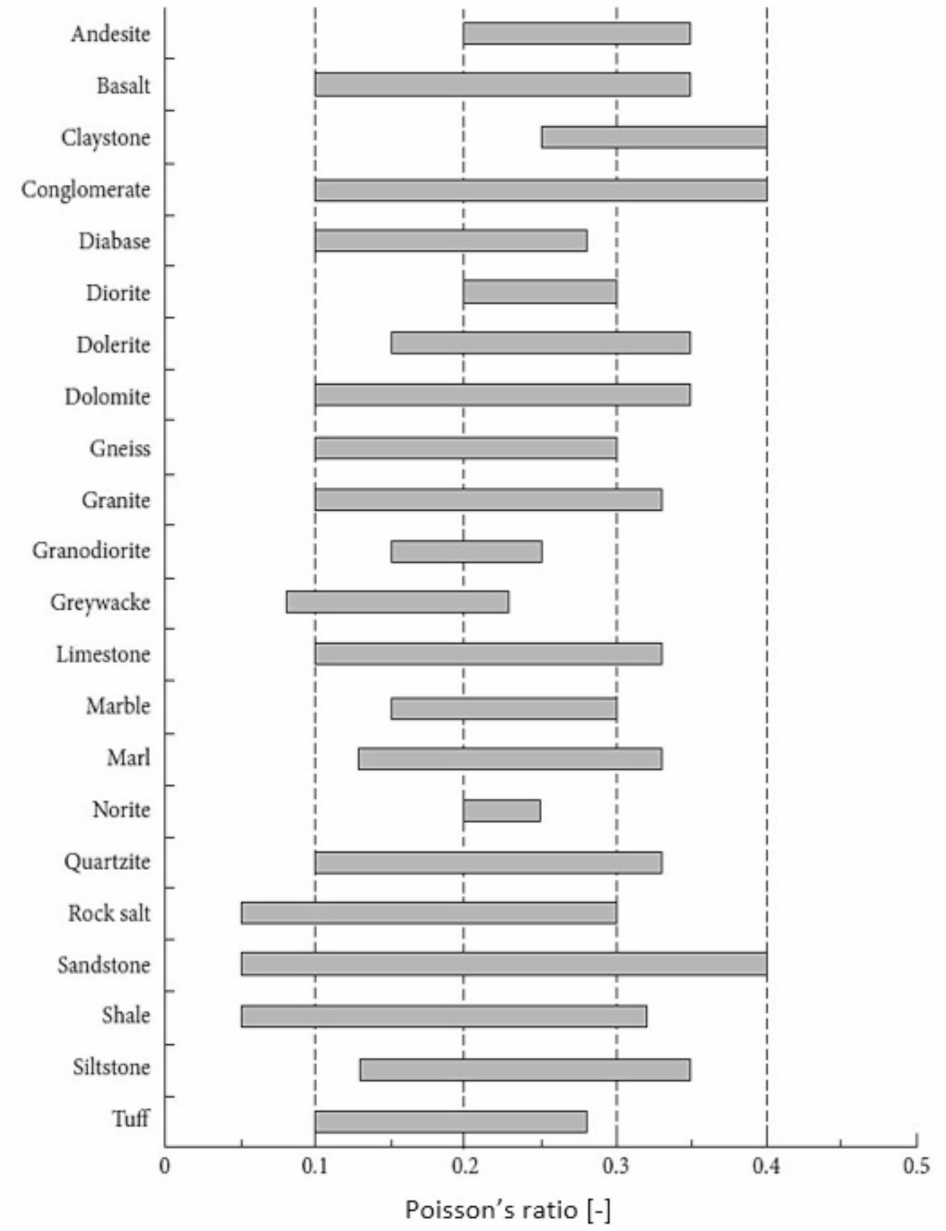
Distribution of Poisson’s ratios for typical types of rocks^2^.

Figure 1 displays the typical range of Poisson’s ratios for some common rocks^2^. Here, it can be seen that the Poisson’s ratios of the majority of rocks demonstrate a wide range, for example, 0.10–0.40 for conglomerate, 0.10–0.33 for limestone, 0.05–0.30 for rock salt, and 0.05–0.40 for sandstone. Due to the wide intervals and the sensitive character of Poisson’s ratio, the presented figure is only informative, but it does not suitable for practical applications without detailed measurements. Even reasonably homogenous materials like marble have a range of Poisson’s ratios where the upper limit (0.30) is twice the lower limit (0.15). Due to major heterogeneities in their geological history, mineral composition, crystallization, or depositional structure, rocks’ Poisson’s ratios can vary widely. Additionally, it is not obvious from the literature the applied testing methodology and if the Poisson’s ratios given are average or secant, although the test and calculation techniques had an impact on the reported Poisson’s ratios.

There is not as much available research on the behavior of the Poisson’s ratio in each step of rock deformation as there are on other factors like Young’s modulus and compressive strength. Research spanning decades reveals the intricate dynamics of Poisson’s ratio in rocks. Walsh^4^ attributed its fluctuations to the cyclic opening and closure of rock cracks, challenging the notion of a constant ratio. Uniaxial compression tests exemplify the continuous ascent of Poisson’s ratio. Notably, the tangent Poisson’s ratio identified by You and Hua^11^ consistently increases, posing challenges in its calculation compared to the elastic modulus. Yu et al.^12^ conducted extensive rock tests, unveiling that the tangent Poisson’s ratio rises under compressive stress and falls during tensile stress. Xing et al.^13^ delved into Poisson’s ratio evolution across various strain rates, revealing its non-dependence on a pure elastic stage. Davarpanah et al.'s^14–16^ studies shed light on the stress-dependent nature of Poisson’s ratio, exploring its relationship with mechanical parameters in Hungarian granitic rocks. Additionally, they proposed empirical relationships between tangent and secant Poisson’s ratio for diverse rock types.

The Poisson’s ratio and the Hoek–Brown material constant (m_i_) a linear connection, according to Vásárhelyi’s^17^ assumption, where the Poisson’s ratio linearly declines as the Hoek–Brown constant (m_i_) is increased:

1 $$\nu = 0.257 - 0.003{\text{m}}_{{\text{i}}}$$

Also, Vásárhelyi and Kovács^18^ presented different empirical methods for calculating the mechanical parameters of rock masses, such as Poisson’s ratio and the Mohr–Coulomb parameters by using the well-known empirical rock mass classification systems, such as RMR, Q and GSI. The published methods, however, still consider Poisson’s ratio as material constant, which depends only on the determined RMR, Q and GSI values of a given rock zone. Moreover, Narimani et al.^19^ indicated that under the assumption of a homogeneous isotropic rock mass, accurate estimation of Poisson’s rock mass ratio relies on the precise estimation of Poisson’s ratio of intact rock and rock mass quality.

According to the studies conducted by Lógó and Vásárhelyi^20^, the Poisson’s ratio exhibits a dependence on the hardness of intact rock. The trend indicates that as rock material transitions to increased brittleness, there is a corresponding decrease in the Poisson’s ratio. Furthermore, their subsequent research later^21^ delved into the impact of variations in the Geological Strength Index (GSI) and environmental pressure on the Poisson’s ratio. Notably, a decrease in the GSI value was associated with an increase in the Poisson’s ratio and heightened environmental stress (σ3) led to a similar elevation in the Poisson’s ratio. In the realm of theoretical relationships, Lógó and Vásárhelyi^22^ introduced a model linking the Poisson’s ratio of intact rock to the confining pressure. This model, grounded in the Hoek–Brown failure criteria, assumed a linear increase in the Poisson’s ratio. Interestingly, they proposed that at the brittle-ductile transition point, the Poisson’s ratio reaches a value of 0.5.

In order to find the axial stress and unload the lateral stress, Xu et al.^23^ suggested a test method that offered a novel way to assess Poisson’s ratio, but it needs advanced test equipment. The tangent, secant, and average Young’s moduli were contrasted in a recent work by Malkowski et al. (2018). According to their research, the tangent Young’s modulus should be used as the guiding parameter at a fixed range of 30–70% of the ultimate load. It is appropriate to refer to the secant Young’s modulus—which ranges from 0 to 50% of the ultimate stress—as the modulus of deformability because it accounts for both elastic strain and pore compaction^24^. The present paper mentions the values of crack initiation stress (σ_ci_) and crack damage stress (σ_cd_), which can be calculated based on the following methods: Onset dilatancy method^25^; Crack volumetric strain method^26^ and change of Poisson’s ratio method^27^.

The objective of this investigation is to assess the changes in Poisson’s ratio for intact granitic rocks throughout the entire span of a Unconfined Compressive Strength (UCS) test. This research introduces an innovative technique to quantify Poisson’s ratio values, spanning from the initial phase to the failure stage. Utilizing raw test data, the study employs three distinct calculation methods: secant, average, and tangent approaches. Through these methodologies, a comprehensive understanding of how Poisson’s ratio evolves during the UCS test is sought, enhancing our insight into the mechanical behavior of intact granitic rocks.

## Methods and results

Since there is no accepted definition for the crack closure stress (σ_cc_) and the crack initiation stress (σ_ci_), they are frequently used as the lower and upper stress limit of the ideally elastic (roughly linear) segment of stress–strain curve instead. For rock materials, σ_ci_ is roughly 30–50% of their uniaxial compressive strength (σ_c_ or UCS) values and σ_cd_ is 60–80% of its UCS. That approach makes possible to determine the average elastic constants (Young’s modulus and Poisson’s ratio) of rocks for the great majority of rock types using the stress–strain curve^28^.

We conducted uniaxial compression tests on Morágy granitic rock formation in Hungary in order to investigate the variation of the Poisson’s ratio by new method and formulation from the crack closure stage though failure stage. The laboratory samples were obtained from research boreholes drilled into the Carboniferous Mórágy Granite Formation during the research and construction phases of a deep geological repository for low and intermediate-level radioactive waste. This granite formation is an intruded and displaced Variscan granite pluton located in South-West Hungary. The primary rock types include microcline megacryst-bearing, medium-grained biotite monzogranites, and quartz monzonites. Spatially, the monzogranitic rocks generally feature oval-shaped, variably elongated monzonite enclaves (mostly amphibole-biotite monzonites, diorites, and syenites) of various sizes (ranging from a few centimeters to several hundred meters), indicating the mixing and mingling of two magmas with different compositions. Additionally, feldspar-quartz rich leucocratic dykes associated with late-stage magmatic evolution, as well as Late Cretaceous trachyte and tephrite dykes, intersect all the previously mentioned rock types. A servo-hydraulic machine under computer control that was configured for continuous load control mode was used for the experiments. Samples were loaded precisely with an accuracy of 0.01 kN at a constant rate of 0.6 kN/s throughout the experiments. Using both axial and lateral strain gauges, the deformations that the samples underwent were measured. It is important to note that the cylindrical rock samples followed the L/D ratio of 2/1, where L represented the sample’s length and D represented its diameter. The rock mechanics lab conducted forty-two uniaxial compressive tests in all. As a result of uniformly distributed axial stress, the Poisson’s ratio is defined as the ratio of the radial strain and the corresponding axial strain^29^:

2 $${\upnu } = - {\text{d}}\upvarepsilon _{{{\text{trans}}}} /{\text{d}}\upvarepsilon _{{{\text{axial}}}}$$

where ε_trans_ denotes the transverse strain, ε_axial_ is the axial strain, and ν is the Poisson’s ratio (positive strain indicates contraction, and negative strain indicates extension). Isotropic and linear elastic materials have a Poisson’s ratio that is, in theory, constant and ranges from -0.5 to + 0.5. It is consistently positive and ranges from 0.05 to 0.40 for rock materials.

**Fig. 2:**
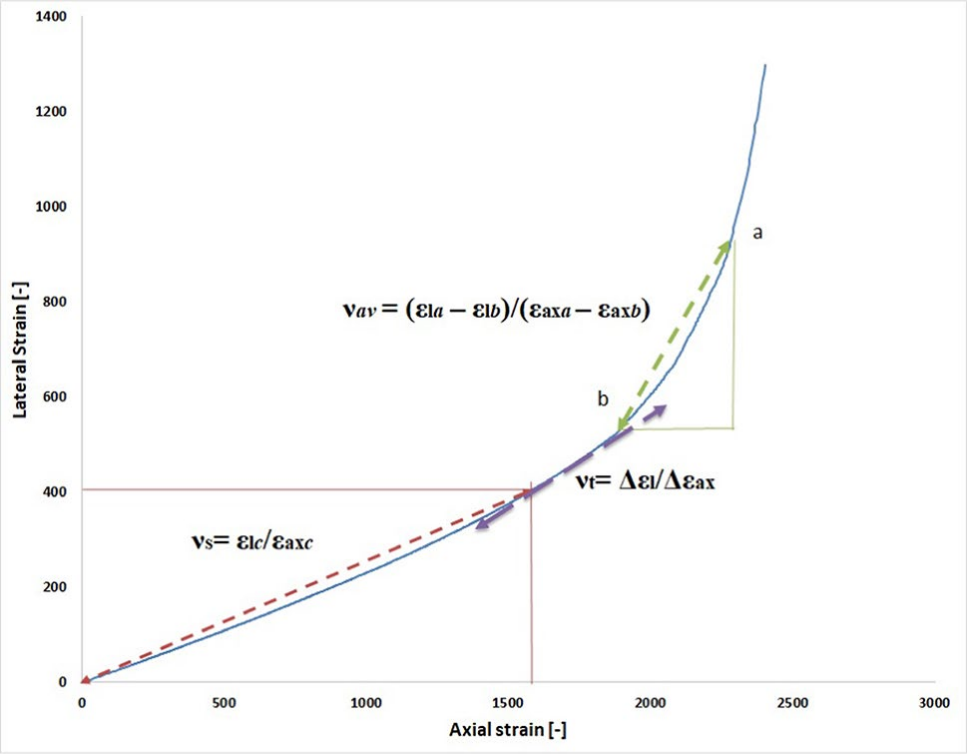
Schematic calculation of secant Poisson’s ratio (ν_s_), tangent Poisson’s ratio (ν_t_) and average Poisson’s ratio (ν_av_)^29^.

Figure 2 displays the equations for calculating the tangent, average, and secant Poisson’s ratios. It should be noted that the lateral strains were regarded as positive values to make it simpler to exhibit their values in the four axial strain-lateral strain curves.

The secant’s origin, initially at zero-stress, shifts with stress (point C in Fig. 2) to analyze its Poisson’s ratio. Phases include low-stress crack closure with minor, unstable lateral strains influenced more by material properties than longitudinal deformation. In this stage, Poisson’s ratios are small, slowly increasing, and variably negative early in loading. The secant Poisson’s ratio linearly grows with axial stress at 20–30% and nonlinearly at 70–80% of peak strength, surpassing typical pre-failure Poisson’s ratio values^1^.

The average Poisson’s ratio reflects axial and radial strain changes within a stress interval. Strains increase as the stress moves from low to high levels. Like secant Poisson’s ratio, lateral strain behavior depends on material properties. Average Poisson’s ratio is prominent in the linear stage, rising proportionately to axial stress at 20–70% of peak value. It expands rapidly and mostly nonlinearly at 80% of peak strength, similar to the secant case.

**Fig. 3:**
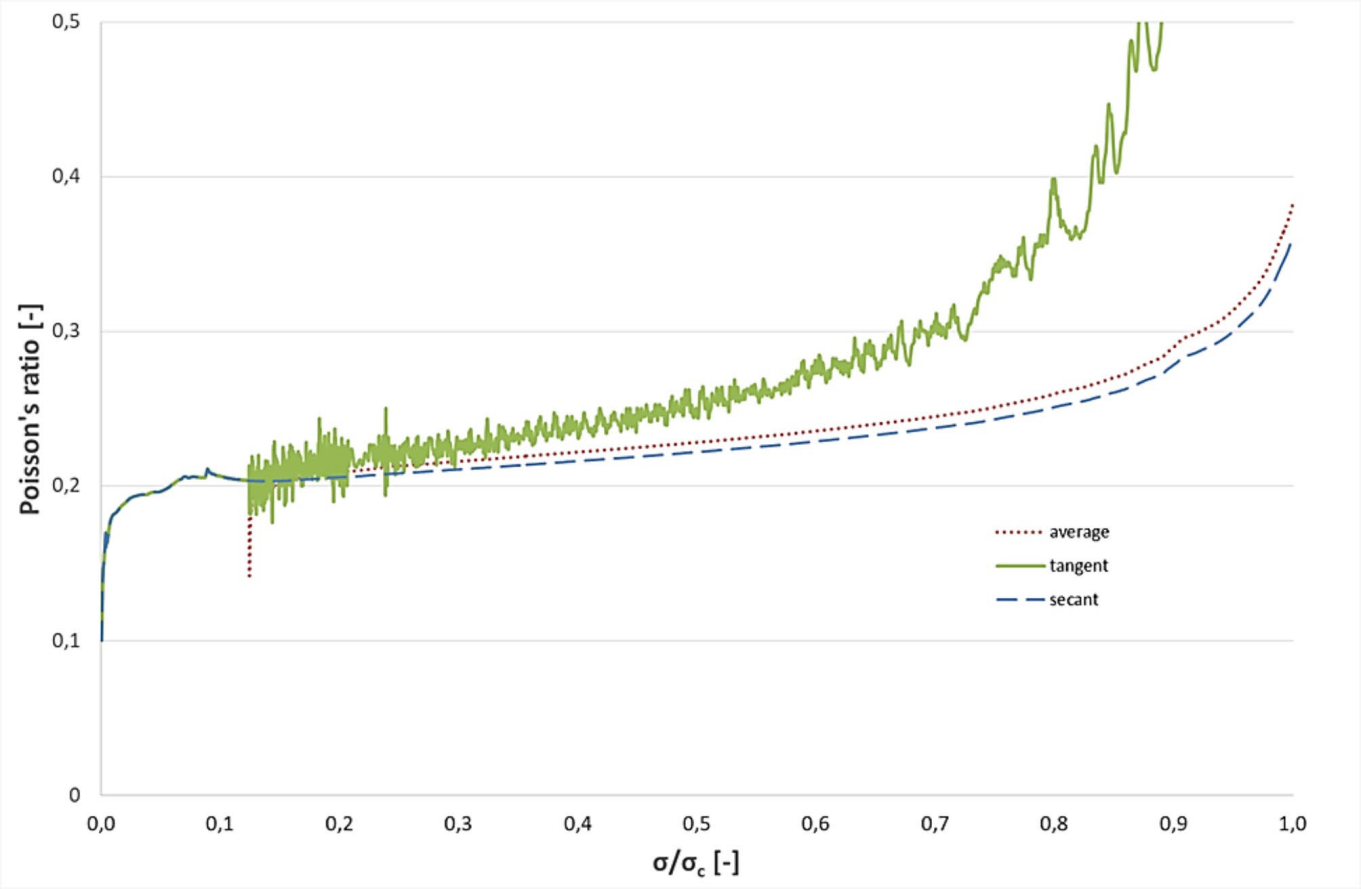
A typical secant, tangent and average Poisson’s ratio in function of (σ/σ_c_) curve for granitic specimen^30^.

The tangent Poisson’s ratio, derived from the axial strain-lateral strain curve’s slope, is sensitive to testing variations and sample frequency compared to secant Poisson’s ratio. Knowledge of the sample interval, determined by stress requirements, is crucial for calculating the tangent Poisson’s ratio. Its value and curve shape depend on the stress interval length, influenced by artificial preparation and data selection procedures. Various preprocessing techniques yield different values, impacting parameter objectivity. Method uncertainties affect rock deformation analysis, as shown in typical Poisson’s ratio vs. σ/σc curves for granitic specimens (Fig. 3)^30^.

**Fig. 4:**
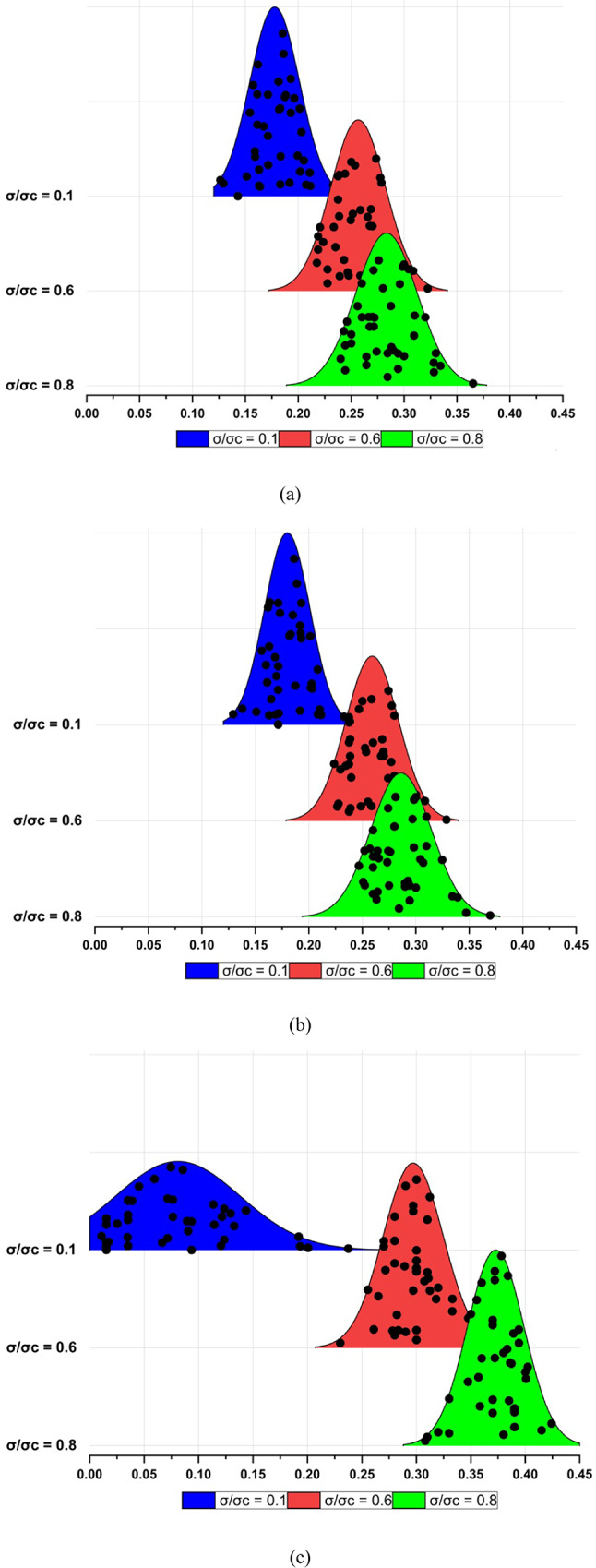
Histogram of Poisson’s ratio for σ/σ_c_ = 0.1, 0.6 and 0.8 in three different scenarios, (**a**) secant, (**b**) average and (**c**) tangent.

To comprehend the frequency distribution of Poisson’s ratio values and discern the intricate relationships among parameters, an in-depth data analysis was conducted using SPSS software^31^. This comprehensive study involved rigorous reliability assessments and the examination of 42 granitic rocks. In three distinct scenarios, Fig. 4 vividly illustrates the frequency distribution of Poisson’s ratio values at σ/σc ratios of 0.1, 0.6, and 0.8.

The exploration of various Poisson’s ratio components revealed intriguing patterns. The secant Poisson’s ratio (ν_sec_) exhibited a range between 0.126 for crack closure and 0.365 for crack damage, while the average Poisson’s ratio (ν_ave_) fluctuated between 0.129 for crack closure and 0.370 for crack damage. Simultaneously, the tangent Poisson’s ratio (ν_tan_) showcased a variation from 0.011 for crack closure to 0.414 for crack damage.

The analysis of the frequency distribution underscored a noteworthy observation—the dispersion of values was more pronounced for secant and average Poisson’s ratios compared to the tangent Poisson’s ratio values. Further insights into the statistical nuances are indicated in Table 1, presenting a meticulous examination of data for all 42 granitic rock samples across the stages of crack closure (cc), crack initiation (ci), and crack damage (cd) concerning secant, average, and tangent Poisson’s ratio.

**Table 1:** Statistical analysis of Poisson’s ratio of 42 granitic rock samples in cc, ci and cd points.

	Maximum			Minimum			Mean			Standard deviation		
σ/σ_c_	0.1	0.6	0.8	0.1	0.6	0.8	0.1	0.6	0.8	0.1	0.6	0.8
Secant Poisson’s ratio	0.233	0.322	0.365	0.126	0.218	0.240	0.177	0.256	0.283	0.024	0.026	0.029
Average Poisson’s ratio	0.233	0.329	0.370	0.129	0.224	0.247	0.179	0.259	0.286	0.021	0.025	0.028
Tangent Poisson’s ratio	0.237	0.377	0.414	0.011	0.230	0.308	0.079	0.297	0.373	0.058	0.027	0.026

In the presented study, a novel approach was employed to examine the evolution of Poisson ratio values throughout the entire process, spanning from crack initiation to the ultimate failure phase within the UCS test. Employing this innovative methodology, the stress range relative to the peak stress (σ/σc) was explored, encompassing a spectrum from -80 to + 80, representing the extremes of σ/σc = 0 and 1, respectively. To achieve symmetry, the coordinate system’s origin for all specimens was shifted to σ/σc = 0.5. Subsequently, a newly proposed model was intricately aligned with the experimental data, fitting seamlessly to the graph through the application of Eq. 3.

3 $$\nu = \nu_{0} + {\text{tan}}\left( {{\text{degree}}} \right)/{\text{B}}$$

In the mathematical expression, the symbol ν_0_ signifies the Poisson’s ratio corresponding to σ/σ_c_ = 0.5, represented as the unchanging constant A. In the context of tangent values measured in degrees, the introduction of the degree variable emerges due to the fact that tan90 yields infinity. This degree is expressed as 160σ/σc-80. The constant B, inherent to the equation, is contingent upon the specific rock type under consideration. Consequently, the ultimate formulation for determining Poisson’s ratio unfolds in the subsequent manner:

4 $$\nu = {\text{A}} + {\text{tan}}({16}0{{\varvec{\upsigma}}}/{{\varvec{\upsigma}}}_{{\mathbf{c}}} - {8}0)/{\text{B}}$$

In pursuit of this objective, Poisson’s ratio calculations were conducted employing a recently introduced model across three distinct scenarios. These scenarios encompassed the examination of Poisson’s ratio through secant, average, and tangent perspectives, with constants A and B uniquely determined for each scenario. The derived results from these calculations have been succinctly compiled and presented in the form of a comprehensive summary in Table 2. Additionally, Table 3 highlights the intricate statistical details by showcasing the mean and standard deviation of constants A and B derived from data collected from all 42 granitic rock samples. This data encompasses various scenarios such as secant, average, and tangent Poisson’s ratio. Thus, in similar circumstances, the provided equations can be applied more effectively.

**Table 2:** Equations and related constants for Poisson’s ratio in different cases.

Poisson’s ratio	Equation	Constant A	Constant B
Secant	ν_sec_ = A_sec_ + Tan (160**σ/σ**_**c**_-80)/B_sec_	0.21–0.30	25–40
Average	ν_ave_ = A_av_ + Tan (160**σ/σ**_**c**_-80)/B_av3_	0.22–0.31	22–40
Tangent	ν_tan_ = A_tan_ + Tan (160**σ/σ**_**c**_-80)/B_tan_	0.22–0.34	9–20

**Table 3:** Statistical analysis of the constants A and B in different cases.

Poisson’s ratio	Constant A			Constant B		
	Range	Mean	Std.Dev	Range	Mean	Std.Dev
Secant	0.21–0.30	0.25	0.02	25–40	35	5.8
Average	0.22–0.31	0.25	0.02	22–40	34	5.72
Tangent	0.22–0.34	0.27	0.03	9–20	13	2.21

**Fig. 5:**
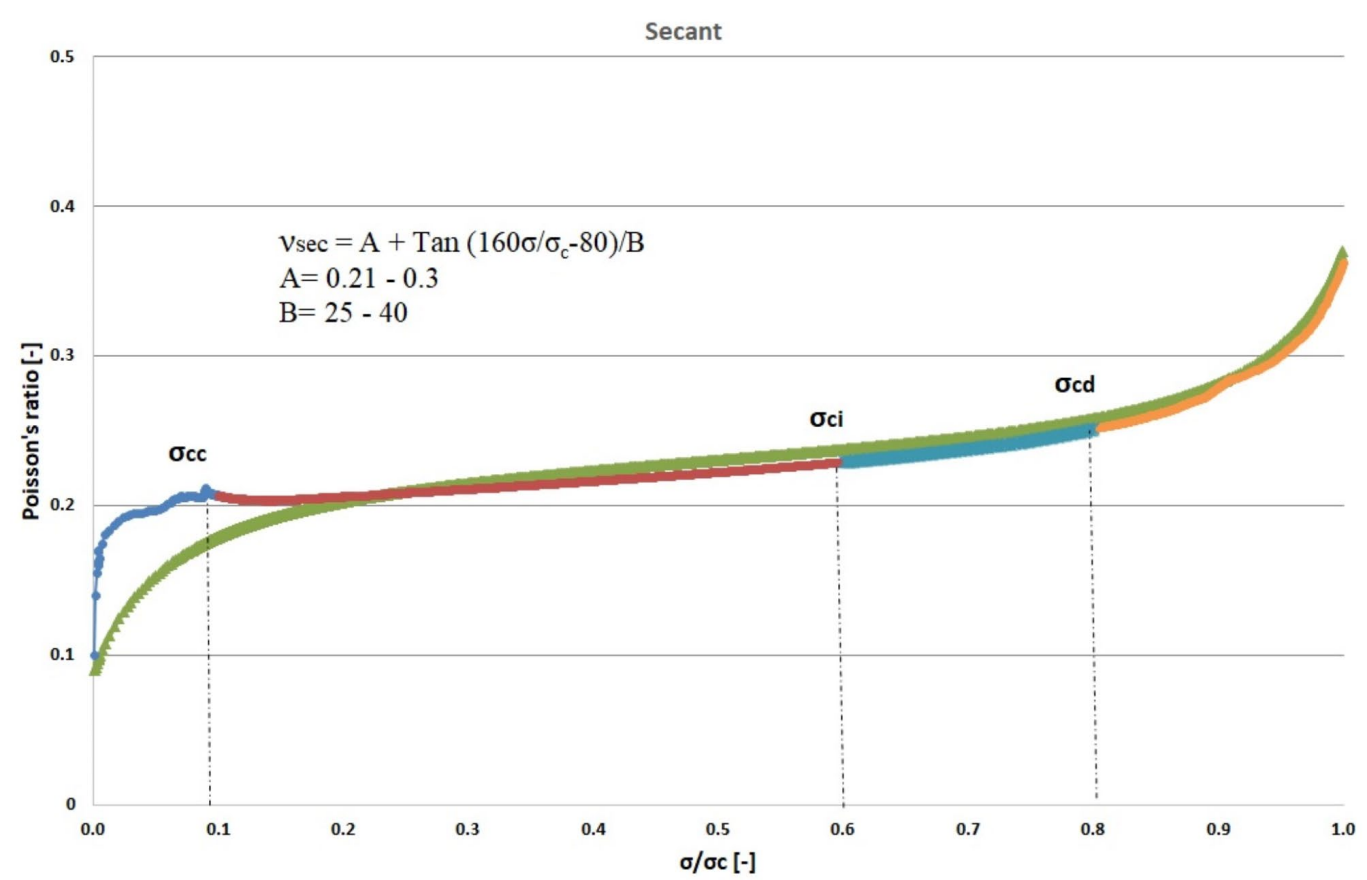
Secant Poisson’s ratio- σ/σ_c_ curve for granitic specimen.

**Fig. 6:**
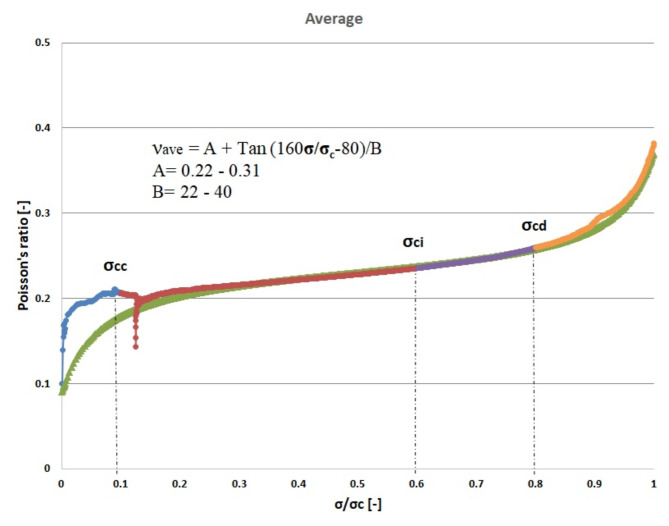
Average Poisson’s ratio- σ/σ_c_ curve for granitic specimen.

**Fig. 7:**
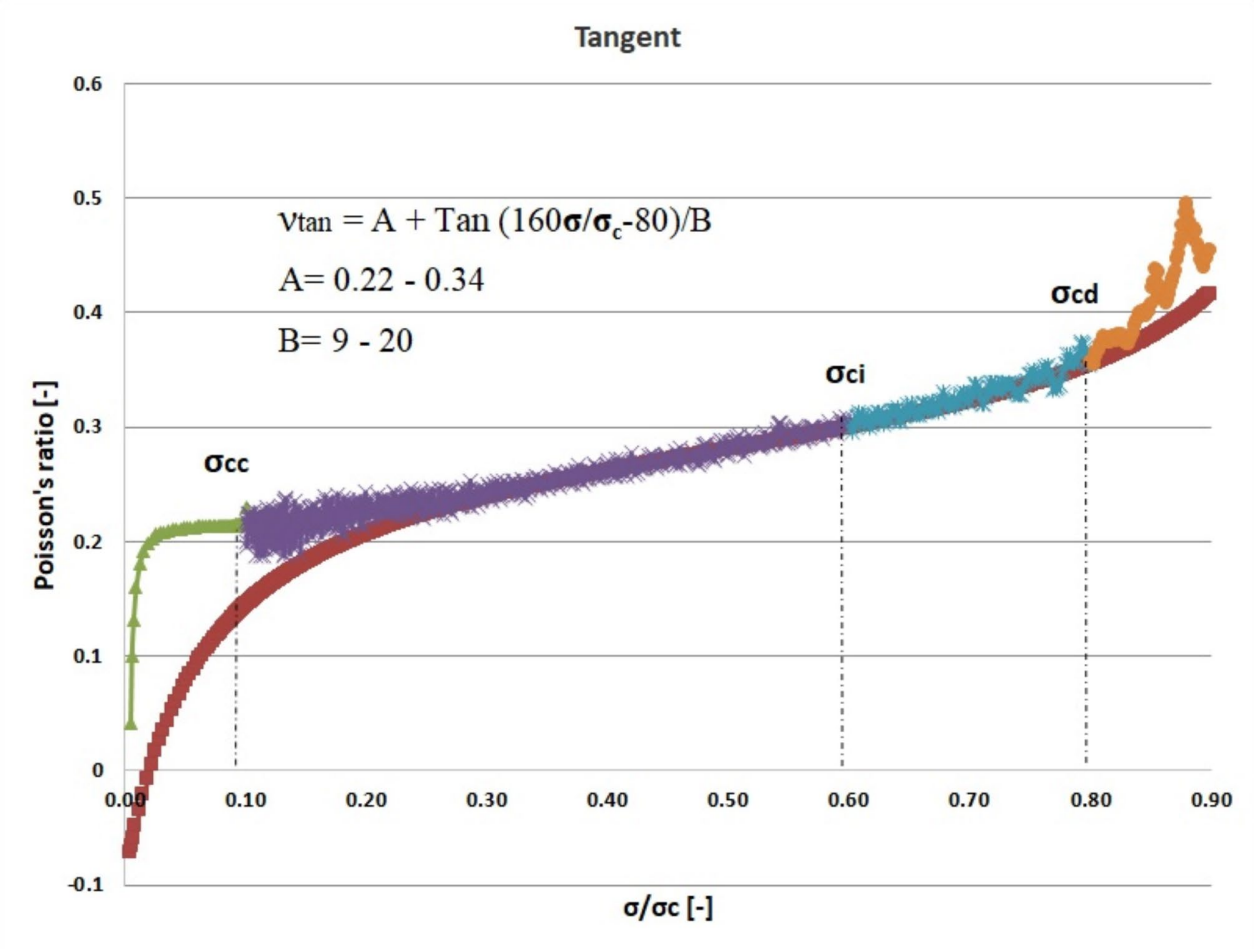
Tangent Poisson’s ratio- σ/σ_c_ curve for granitic specimen.

Figures 5, 6, and 7 illustrate the dynamic fluctuations in Poisson’s ratio concerning σ/σ_c_, encompassing secant, average, and tangent scenarios. The visual representations indicate a steeper slope in the tangent function, showcasing a more pronounced variation in Poisson’s ratio compared to the secant and average counterparts. As per the proposed model, the optimal alignment between our theoretical framework and experimental data focuses within the spectrum spanning crack closure to the stress stages associated with crack damage. Additionally, the secant and average Poisson’s ratios demonstrate an almost linear progression as stress fluctuates between the phases of crack closure and the onset of damage during the loading process.

## Quantitative relationships and errors

**Fig. 8:**
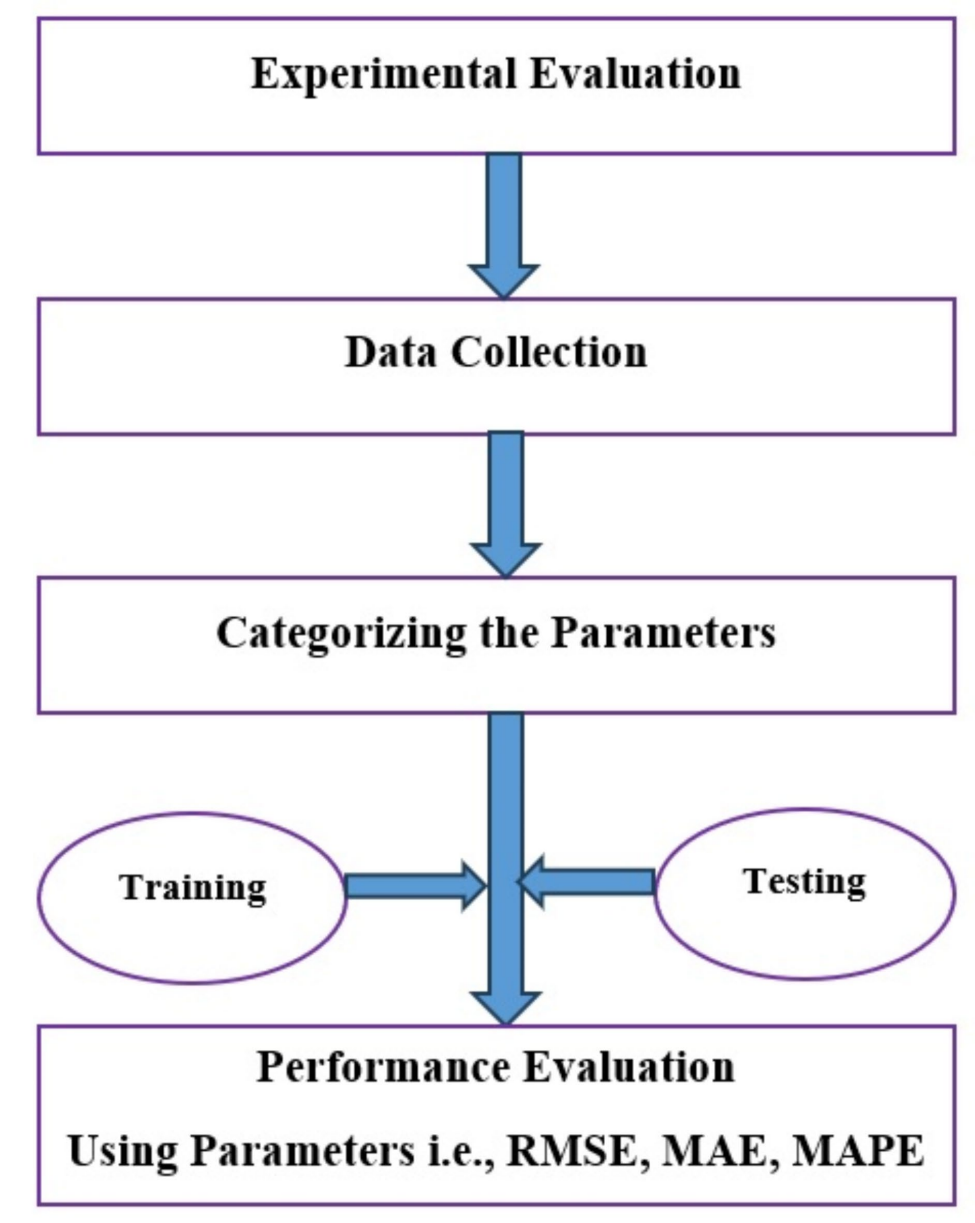
The implemented proposed method flowchart.

The obtained database, which contained 42 data points relating to the testing and determination of the geomechanical features, served as the basic information set to illustrate the use of the proposed method. As a result, the database was split into a training set and a test set. 80% of the main database was in the training set, while the remaining 20% was in the test set. Prior to being assessed on the test set, the proposed method was first applied to the training set. Figure 8 displays the proposed equation’s structural flowchart. Following instruction, the new technique was tried on the test set while being evaluated against the evaluation criterion.

The statistical effectiveness of the Poisson’s ratio prediction was evaluated using four widely used statistical measures, including determination coefficient (R^2^), root mean square error (RMSE), mean absolute error (MAE), and mean absolute percent error (MAPE) in the training and testing sets. The following performance measurements are defined by Eqs. (5) through (7).

5 $${\text{RMSE}} = \sqrt {\frac{{\mathop \sum \nolimits_{{{\text{i}} = 1}}^{{\text{n}}} \left( {{\text{pi}} - {\text{qi}}} \right)^{2} }}{{\text{n}}}}$$

6 $${\text{MAPE}} = \frac{1}{{\text{n}}}\mathop \sum \limits_{{{\text{i}} = 1}}^{{\text{n}}} \left| {\frac{{{\text{qi}} - {\text{pi}}}}{{{\text{qi}}}}} \right| \times 100$$

7 $${\text{MAE}} = \frac{{\mathop \sum \nolimits_{{{\text{i}} = 1}}^{{\text{n}}} \left| {{\text{pi}} - {\text{qi}}} \right|}}{{\text{n}}}$$

Here, n stands for the total number of experiments, while pi and qi represent the i-th projected and expected findings, respectively. RMSE is a widely used metric because significant errors are handled with much more successfully than minor ones. The prediction error was minimal when the RMSE was close to zero. It does not, however, always ensure excellence in performance. Additionally, MAE was determined, which is very useful when the data is continuous and smooth. The proposed equation would be excellent if RMSE = 0, MAE = 0 and MAPE = 0. Table 4 lists these new statistical indicators that offer quantifiable relationships between Poisson’s ratio in various situations and values.

**Table 4:** Statistical indicators for Poisson’s ratio of granitic rocks in different scenarios.

Statistical metrics	Secant Poisson’s ratio						Average Poisson’s ratio						Tangent Poisson’s ratio					
	Training			Testing			Training			Testing			Training			Testing		
σ/σ_c_	0.1	0.6	0.8	0.1	0.6	0.8	0.1	0.6	0.8	0.1	0.6	0.8	0.1	0.6	0.8	0.1	0.6	0.8
RMSE	0.03	0	0	0.04	0	0	0.04	0	0.01	0.04	0	0.01	0.03	0	0.01	0.04	0	0.01
MAE	0.03	0	0	0.04	0	0	0.03	0	0	0.04	0	0	0.03	0	0	0.04	0	0
MAPE (%)	15	0.8	1.3	18	0.6	1	15	1.3	1.7	17	1	2	15	1	1.7	17	1	2

**Fig. 9:**
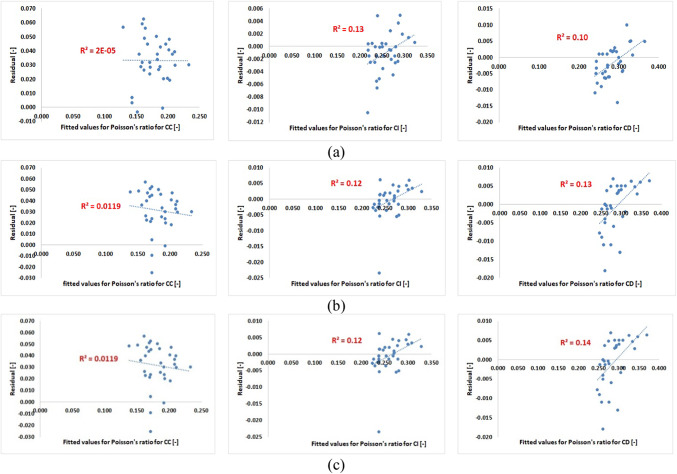
Distribution of the residuals against fitted values (**a**) secant Poisson’s ratio, (**b**) average Poisson’s ratio, (**c**) tangent Poisson’s ratio.

The suggested model’s error can be determined by plotting the residuals against the fitted values for the dependent variable. The differences between experimental results and values anticipated by the proposed model are known as residuals. The proposed model’s predictions match the values that have been fitted. The residuals’ symmetrical and balanced dispersion about the horizontal axis is seen in Fig. 9. The residuals’ independent nature and random distribution around the centerline are confirmed by this distribution. To make sure of this, the determination coefficient, R-squared, is computed. It turns out to be a very small amount. In other words, almost the residuals are not dependent on one another. The proposed model’s goodness of fit is therefore admirable.

**Fig. 10:**
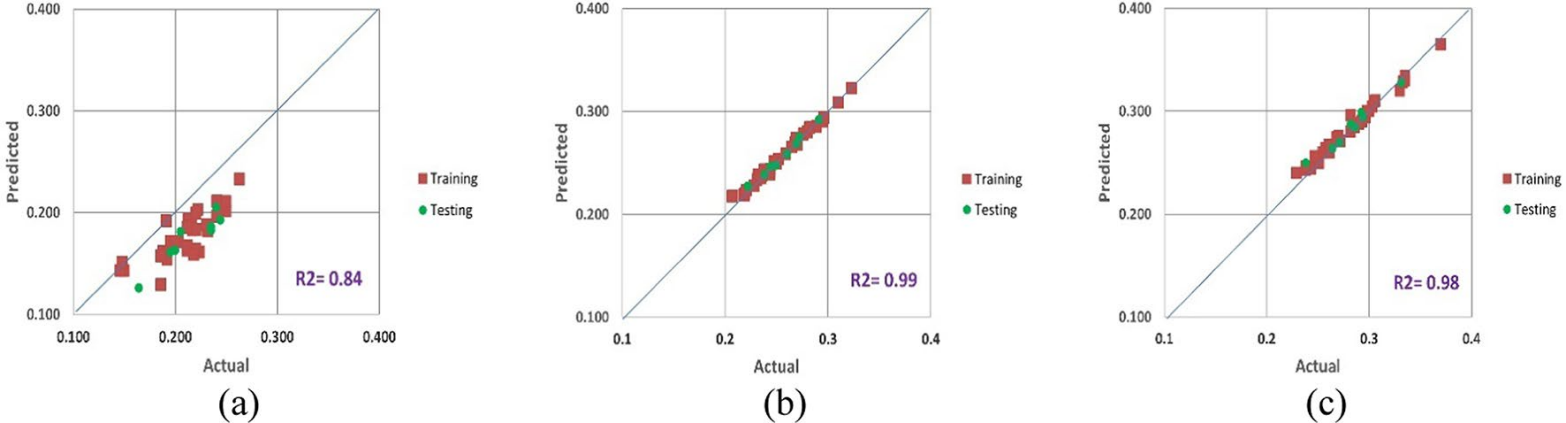
New proposed secant Poisson’s ratio model results for (**a**) CC, (**b**) CI and (**c**) CD.

**Fig. 11:**
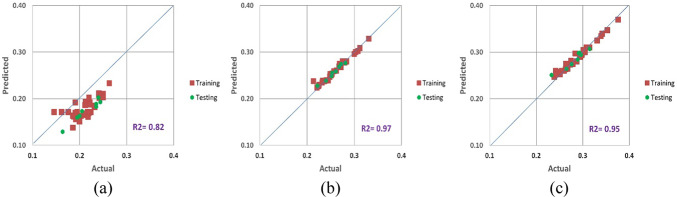
New proposed average Poisson’s ratio model results for (**a**) CC, (**b**) CI and (**c**) CD.

**Fig. 12:**
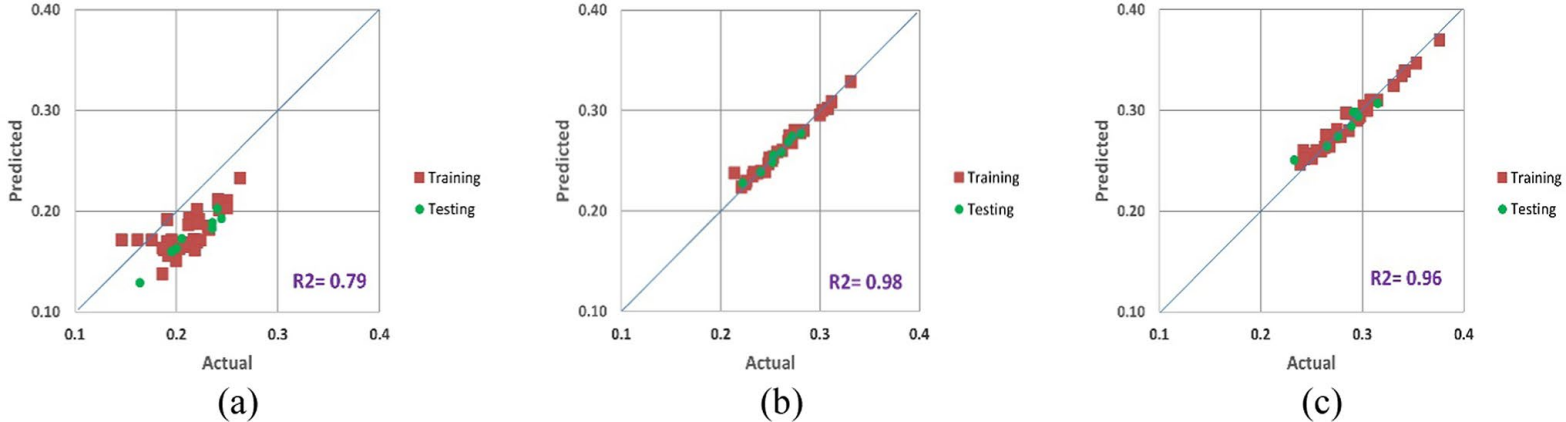
New proposed tangent Poisson’s ratio model results for (**a**) CC, (**b**) CI and (**c**) CD.

Figures 10, 11 and 12 show a contrast between the Poisson’s ratio values that were seen during the training and testing stages and those that were anticipated for three different scenarios. These results aptly show the exceptional ability of the proposed equation to forecast Poisson’s ratio with remarkable accuracy. Notably, the suggested equation’s predictions during the training phase obtained outstanding values for RMSE, MAE, and MAPE for testing as well.

When comparing the predicted results produced by the new equations with the measured values using regression analysis, the experimental findings were used as comparative elements to evaluate the performance of the proposed technique. The variation of the dependent variable that was predicted by the independent variables was estimated using the coefficient of determination (R^2^). R^2^, which typically varies from 0 to 1, represents the relationship between the observed outcomes and the observed predictor value. The closer R^2^ is to 1, the more accurate the data overlap is. According to Figs. 10, 11 and 12, the estimated R^2^ for the Poisson’s ratio in all the scenarios (secant, average and tangent) are high for the crack initiation and crack damage phases, but less for the crack closure phase.

## Discussion

The objective of this theoretical investigation is to establish a straightforward mathematical formulation between σ/σ_c_ and intact rock’s Poisson’s ratio value. Considering the dynamic nature of a rock’s Poisson’s ratio, the conventional approach of treating it as a constant in the linear elastic deformation stage becomes dubious. Poisson’s ratio exhibits continuous variations, challenging the notion of its static nature. The implications of these fluctuations extend to the intricate division of progressive failure processes. The value of Poisson’s ratio proves to be a pivotal factor, exerting substantial influence on the categorization of failure stages. Remarkably, even a minor fluctuation of ± 0.05 in Poisson’s ratio can induce a profound impact, altering the crack propagation threshold by more than 40%^32^. The variation of Poisson’s ratio of the 42 granitic rocks from Bátaapáti radioactive waste repository (Hungary) was calculated by using the new formula from the beginning of loading till failure stage at UCS test. As shown in Table 2 and Figs. 5, 6 and 7, it can be more accurately described as an elastic deformation parameter that monotonically increases with stress from the crack closure stage to the crack damage stage of the compressive processes; however, the increase in Poisson’s ratio from the beginning of the loading up to the crack closure and from crack damage stage up to the failure is steeper. The validity of the equation for estimating the Poisson’s ratio is studied by comparing the performances of the newly proposed mathematical equation utilizing the findings of accuracy measures, root mean square error (RMSE), mean absolute error (MAE), and mean absolute percent error (MAPE). The independent variables of the training data were examined after the data were first randomly divided into training and test sets in an 80:20 ratio (80% training and 20% testing). After the prediction of the new mathematical equation, the results are compared to the actual data of the Poisson’s ratio for the crack closure, crack initiation and crack damage points in three different scenarios (secant, average and tangent), as shown in Table 4 and Figs. 9, 10 and 11 and the results indicate very close relationship based on RMSE, MAE and MAPE. The pairwise relationships among predicted Poisson’s ratio and the actual values express high accordance for all the points, although they are more reliable for the crack initiation and crack damage in all the scenarios.

Examining the gradual failure of a granite specimen, Dong et al.^1^ discovered that in hard rock formations, both the secant and average Poisson’s ratios exhibit a nearly linear increase with stress during the primary phases of the loading process, aligning with the observations of the present study. However, non-linear variations are evident before crack closure and following crack damage, indicating complex behaviors in these specific stages. Moreover, in Dong et al.'s research^1^, they examined the fluctuation of the tangent Poisson’s ratio concerning the stress interval width, focusing on both marble and sandstone rocks. The findings highlight that when the stress interval width is approximately 1% of the Unconfined Compressive Strength (UCS), the estimated tangent Poisson’s ratios exhibit substantial oscillations^33^. As the stress interval widens, the fluctuation diminishes, reaching stability at intervals of 5% of UCS or higher. Notably, the testing perspective reveals that the tangent Poisson’s ratio essentially represents the average Poisson’s ratio within a confined stress interval, leading to unreliable calculations^1^. In contrast, the novel approach introduced in this study allows for the calculation of tangent Poisson’s ratio variations throughout various stages of the Unconfined Compressive Strength (UCS) test. Also, in confirming the robustness of our model, we cross-checked its outcomes against those reported by Davarpanah et al. (2019 and 2020). Our model successfully estimated secant, average, and tangent Poisson’s ratios, revealing admirable alignment with the results presented in their studies.

The present study’s findings thus suggest that the proposed mathematical equation can be effectively utilized to predict the Poisson’s ratio of rocks. However, given that the mechanical behavior of rocks varies depending on their type, the results of this research are valid and feasible for granitic rocks. Accordingly, it is advised that a mathematical method similar to this one be applied for other rock types, so that this method can be developed for different rocks and other geotechnical properties. Moreover, larger databases in empirical modeling enhance generalization capacity and prediction accuracy. Consequently, more research is required to verify the effectiveness of the proposed method of this study.

## Conclusions

This study suggests a novel method for determining the Poisson’s ratio of granitic intact rocks from the fracture closure stage until failure. The main conclusions are as follows:

- Our findings show that the Poisson’s ratio of the examined samples reflects various behaviors. Thus, the results of our research lead to the development of new equations that give accurate estimates of the Poisson’s ratio during loading in the UCS test for secant, tangent, and average cases.
- According to our findings, the values of Poisson’s ratio in all the scenarios for the granitic rocks follow nonlinear function.
- The effectiveness of the suggested strategy was assessed using predicted and actual ʋ values of granitic rocks. When the ʋ values predicted by this function were contrasted with the actual ʋ values, the R^2^ values were discovered to be accurate. According to Figs. 10, 11 and 12, the cross-correlation’s R^2^ values demonstrate that the equation is completely capable of making predictions of Poisson’s ratio.
- The findings demonstrated that the suggested equation worked the best, having the lowest Root Mean Square Error (ranging from 0 to 0.04), the lowest Mean Absolute Error (ranging from 0 to 0.04) and the lowest mean absolute percent error (ranging from 0.6% to 18%). Therefore, the recommended method is shown to have the best fit to experimental data in all scenarios for granitic rocks, consistent with the data, within the range of crack closure via crack damage stress stages.
- The secant Poisson’s ratio develops essentially linearly with changes in stress between the loading process stages of crack closure and crack damage. However, the tangent Poisson’s ratio doesn’t follow this rule.
- The recommended method’s equipment and specimen requirements are the same as those of the conventional approach, and the test process is straightforward. The suggested approach has promising application possibilities and will assist us in accurately determining the fundamental elastic constants of granitic rocks.

The purpose of this study was to evaluate the precision and dependability of this approach for granitic rock Poisson’s ratio prediction. Despite, we want to emphasize that the strategy described here is by no means exhaustive. More laboratory data are now required, along with a more efficient method of accounting for Poisson’s ratio. So, as future work, we plan to investigate the use of different rock types to develop and refine the method for predicting the Poisson’s ratio of rocks. This could potentially improve the accuracy and performance of this method.

## Data Availability

The datasets used and/or analyzed during the current study available from the corresponding author on reasonable request.
